# Integrating survival analysis and machine learning for modeling overtaking duration on rural two-lane highways

**DOI:** 10.1371/journal.pone.0357092

**Published:** 2026-09-01

**Authors:** Chuanhe Shi, Shuai Fu, Yuanchang Zhao, Jing He, Peng Ye, Wenchen Yang

**Affiliations:** 1 Yunnan Key Laboratory of Digital Communications, Yunnan Communications Investment & Construction Group Co., Ltd, Kunming, China; 2 National Engineering Research Center for Efficient Maintenance, Safety and Durability of Roads and Bridges, Broadvision Engineering Consultants Co., Ltd, Kunming, China; National Institute of Technology Calicut, INDIA

## Abstract

Overtaking duration is closely associated with overtaking safety because longer maneuvers increase exposure in the opposing traffic lane. However, evidence on overtaking duration on rural mountainous two-lane highways remains limited, particularly under mixed-traffic conditions. This study investigates the determinants of overtaking duration on rural mountainous two-lane highways using an explainable survival machine-learning framework. Unmanned aerial vehicle videos were collected, and vehicle trajectories were extracted to obtain detailed overtaking maneuver data, including complete and right-censored observations. To model censored duration data and capture nonlinear relationships between overtaking duration and its influencing factors, a random survival forest (RSF) model was developed and compared with a conventional log-logistic accelerated failure time (AFT) model. The results show the average values of overtaking duration and overtaking distance were 10.2 seconds and 201.2 meters, respectively. The RSF model achieved a higher C-index than the log-logistic AFT model, indicating better discrimination of overtaking completion times. SHAP results identified the initial speed of the overtaking vehicle, lateral distance, initial speed difference, and overtaking vehicle type as the most influential factors, with pronounced nonlinear effects observed for overtaking-vehicle speed and speed difference. These findings highlight the potential of interpretable survival machine learning for analyzing overtaking-duration data and provide insights into overtaking behavior and safety on rural mountainous two-lane highways.

## 1. Introduction

China has experienced a marked expansion of its highway system over the past decade, accompanied by increasing network integration. Under the national functional classification, highways are grouped into expressways, first-class highways, second-class highways, and third-class and lower-class highways. As of end-2021, the country’s road network extended to 5,280,700 km, of which 4,557,100 km were two-lane facilities on second-class and lower-class highways, accounting for approximately 86% of the total. In rural mountainous areas, these two-lane highways carry a large proportion of passenger and freight traffic. These roads are typically characterized by no median separation, limited sight distance, steep grades, and small-radius curves. Traffic flow on these roads often consists of passenger cars, heavy vehicles, motorcycles, tractors, and other slow-moving vehicles. Under such conditions, speed differences among vehicles frequently lead to overtaking maneuvers, which require temporary use of the opposing lane and may increase exposure to severe conflicts with oncoming vehicles.

Although overtaking-related crashes are relatively infrequent compared with other crash types, such crashes are often associated with severe injury outcomes. On rural two-lane roads in Iran, Shariat-Mohaymany et al. found that overtaking-related crashes accounted for approximately one-fifth of all crashes but contributed to about 30% of injuries and as much as 50% of fatalities [1]. In Germany, Richter et al. similarly showed that overtaking was involved in roughly 6% of 73,916 reported crashes, yet these crashes accounted for about 9% of casualties [2]. In China, almost 30% of accidents on two-lane roads in rural mountainous areas are associated with overtaking [3]. Therefore, understanding overtaking behavior is important for improving traffic safety on rural two-lane highways.

Overtaking duration and overtaking distance are key indicators of overtaking maneuvers because these two measures directly reflect the maneuver’s exposure window during which the overtaking vehicle occupies or interacts with the opposing lane and have been used in passing-zone risk appraisal [4,5]. Previous studies have examined overtaking duration and distance in different traffic environments. Hegeman et al. collected overtaking maneuvers using an instrumented vehicle and found a mean overtaking duration of 7.8 s under homogeneous traffic flow conditions in the Netherlands [6]. Choudhari et al. identified overtaking duration and distance as 10.0 s and 173.1 m, respectively, under mixed-traffic conditions on India’s two-lane highways [7]. Other studies further showed that overtaking duration and distance exhibit considerable spatial heterogeneity across different roadway environments and traffic conditions [8,9]. This spatial heterogeneity suggests that findings from developed regions or non-mountainous road environments may not be directly transferable to rural mountainous two-lane highways.

To investigate overtaking behavior, previous studies have employed various data collection approaches, including driving simulators, instrumented vehicles, roadside observations, and trajectory-based field measurements. Farah investigated age and gender differences in overtaking maneuvers using an interactive driving simulator and found that driver characteristics, geometric conditions, and traffic conditions significantly affected overtaking frequency, overtaking duration, following distance, accepted gaps, and desired speeds [10]. Llorca and Farah compared passing behavior observed in the field with that obtained from a driving simulator and reported differences in several passing indicators, including passing duration [11]. Karimi et al. compared passing behavior observed in the field with that obtained from a fixed-base driving simulator and reported that several indicators were broadly comparable, although passing duration was higher in the simulator than in the field because of differences in passing speed and speed difference [12]. Although simulator-based studies provide valuable behavioral insights, field video observations remain necessary for characterizing overtaking maneuvers under real traffic conditions. Hassan et al. used roadside video recordings to extract overtaking time, overtaking distance, and related maneuver variables, and identified several factors affecting overtaking-vehicle speed [13]. With the development of unmanned aerial vehicle (UAV) technology and computer-vision-based trajectory extraction methods, drone-based traffic observation has emerged as an effective approach for capturing vehicle trajectories from an aerial perspective [14,15]. The potential of field trajectory data for characterizing overtaking duration and its influencing factors on rural mountainous two-lane highways under mixed traffic conditions has not been fully explored.

In terms of analytical frameworks for overtaking behavior analysis, early studies mainly employed conventional regression-based approaches to investigate the relationships between overtaking duration and traffic-related variables such as speed, relative speed, and accepted gaps [6,13]. Although these approaches provide useful evidence on factors associated with overtaking behavior, they usually treat overtaking duration as a continuous response variable and do not explicitly account for the time-to-completion nature of an overtaking maneuver or the presence of incomplete observations. To address these limitations, survival analysis approaches have been introduced into overtaking-duration modeling. In this context, survival analysis is used as a time-to-completion modeling framework rather than as a failure-process model. Overtaking duration is modeled as the elapsed time from maneuver initiation to maneuver completion, and incomplete maneuvers observed within the video window can be treated as right-censored observations. For example, Maji et al. developed a hazard-based overtaking duration model for mixed traffic and found that the Log-logistic distribution provided a suitable representation of overtaking duration across different overtaken vehicle classes [16]. Vlahogianni modeled the acceleration and return stages of overtaking as time-to-event processes and found that the Log-logistic specification provided the best fit among the candidate survival models [17]. However, most existing survival-analysis applications in overtaking studies still rely on parametric or semi-parametric model structures, which often require pre-specified distributional assumptions, proportional-hazard assumptions, or simplified functional relationships between covariates and overtaking duration. These assumptions may limit their ability to represent nonlinear effects and complex interactions among traffic and maneuver-related variables. Survival machine-learning models offer a potential solution because they can handle censored duration data while capturing nonlinear effects and complex interactions without strict parametric assumptions. However, their limited interpretability makes it difficult to explain how specific traffic and maneuver-related variables affect overtaking duration. Therefore, an interpretable survival machine-learning framework is needed to balance predictive flexibility and mechanism-oriented explanation.

To address these gaps, this study develops an interpretable survival machine-learning framework for modeling overtaking duration on a rural mountainous two-lane highway using UAV-based trajectory data and traffic monitoring data collected under real-world traffic conditions. The main contributions are threefold. First, field-based evidence on overtaking duration and overtaking distance is provided for mixed traffic conditions in a rural mountainous area, where trajectory-based studies remain limited. Second, overtaking duration is modeled within a survival-analysis framework, allowing incomplete overtaking observations to be incorporated as censored data. Third, random survival forest (RSF) is combined with SHAP analysis to capture nonlinear relationships between traffic and maneuver-related variables and overtaking duration while improving model interpretability. These contributions provide methodological support for overtaking-duration modeling, risk assessment, and safety management on rural two-lane highways.

## 2. Data collection

### 2.1. Study locations

**Fig 1:**
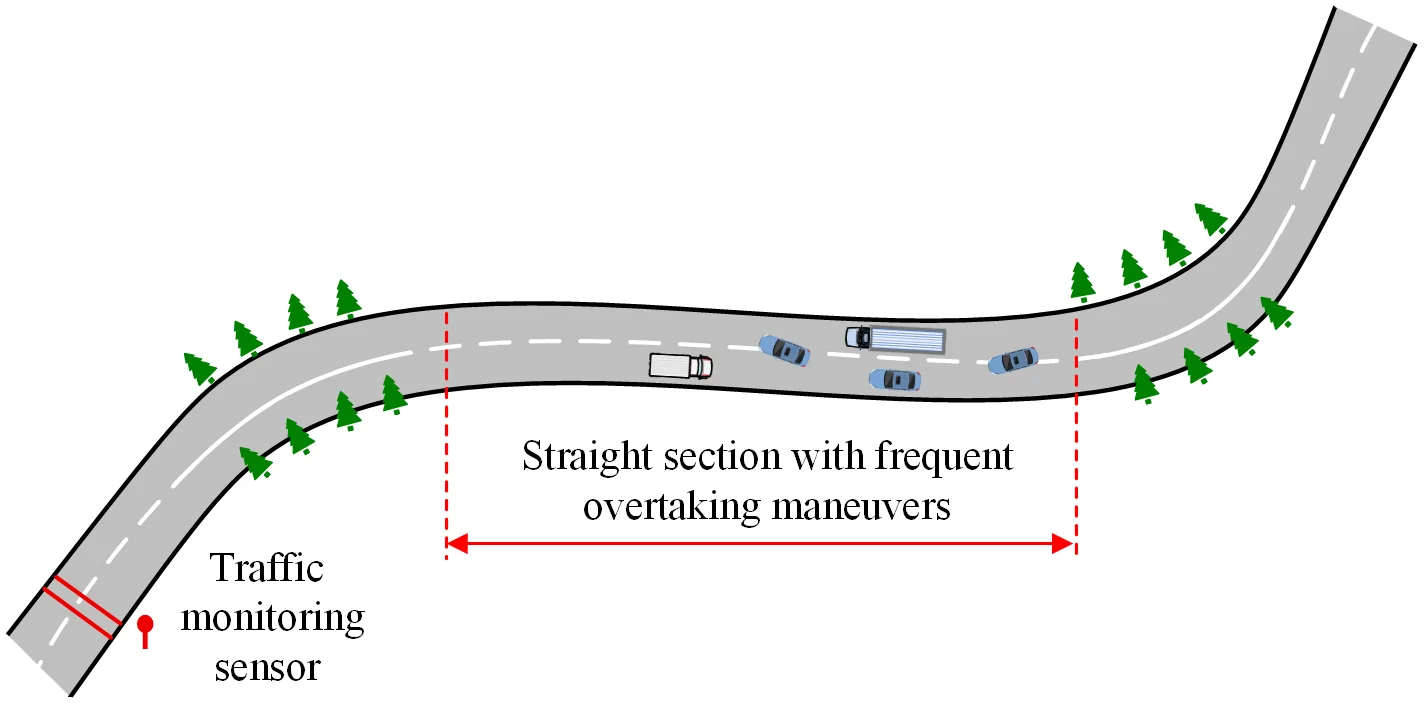
Schematic diagram of the road section with frequent overtaking maneuvers.

Data for this study, including video footage and traffic-flow observations, were collected on Yuan Shuang two-lane highway, a rural mountainous highway in Yunnan Province, China. Overtaking on two-lane highways occurs more frequently on straight sections, whereas overtaking on curved sections entails additional risk due to insufficient sight distance. Therefore, the K81 straight section of Yuan Shuang highway, with a grade of 4.8%, was selected for analysis of overtaking behavior. The schematic diagram of the road section, where overtaking maneuvers were frequently observed, is presented in Fig 1. The design speed is 60 km/h. The total cross-sectional width is 10.5 m, consisting of two 3.75 m lanes and 1.5 m paved shoulders on both sides.

Video data were collected over four days in September 2019, between 08:00 and 18:00 each day. A DJI Air 2S drone was deployed to record high-altitude aerial footage under clear-sky conditions. The flight altitude was maintained at approximately 200 m, covering a road segment of about 300 m in length. Video was acquired at 30 frames per second with a spatial resolution of 3840 × 2160 pixels. The dataset comprises 24 h of recordings. Traffic-flow information, including traffic volume and vehicle type, was collected using a traffic monitoring sensor (MetroCount 5900) installed upstream of the curve near the study section as shown in Fig 1. These data were used to describe the traffic environment during the observation period.

The traffic video and traffic-flow data used in this study were collected on a public rural two-lane highway. No private land, protected area, or restricted field site was accessed during data collection. The UAV was operated only for traffic observation and did not collect personally identifiable information from road users. Therefore, no specific field site access permit was required for this study.

### 2.2. Extraction of overtaking trajectories

The trajectories of overtaking maneuvers were extracted by semi-automated tracking software, Kinovea 8.26. This open-source software has been widely used for motion analysis and road-user trajectory extraction [18]. Kathuria et al. evaluated the accuracy of Kinovea in extracting motion features such as position, velocity, etc [19], and reported the mean relative error, relative precision error and relative accuracy error values of 3.9%, 4.1% and 2.7%, respectively.

**Fig 2:**
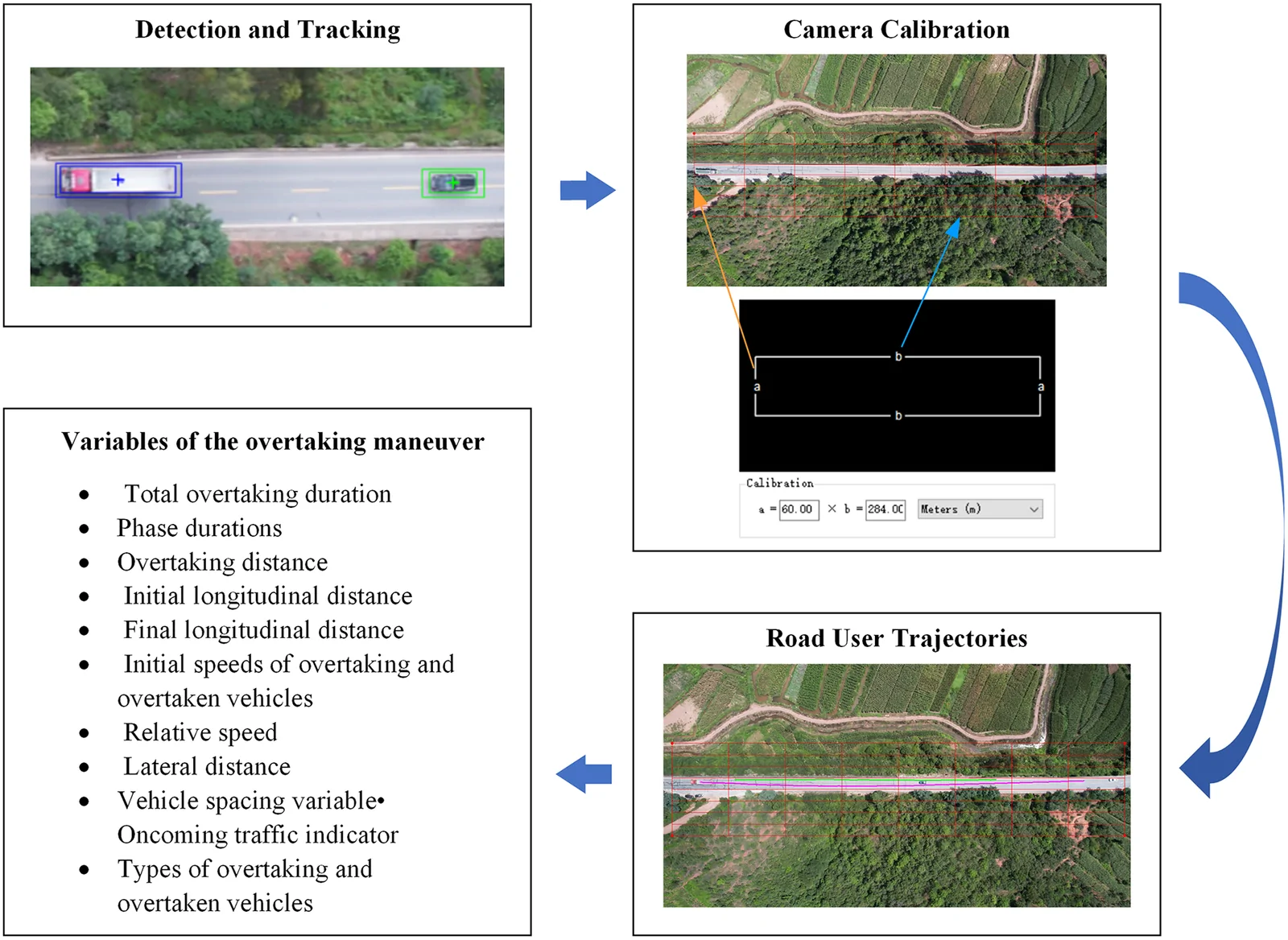
Workflow of overtaking trajectory extraction.

The trajectory extraction procedure is presented in Fig 2. Overtaking video clips were first selected from the UAV recordings, and the relevant vehicles were identified and tracked. A two-dimensional coordinate system was then established based on the road section, and fixed reference points with known distances were used to transform image coordinates into real-world coordinates. After calibration, vehicle trajectories were generated, and overtaking-related variables were extracted.

To improve trajectory quality, the raw trajectories were visually inspected and reconstructed before analysis. Abnormal trajectory points were screened using kinematic threshold criteria, corrected by interpolation, and smoothed before deriving speed and distance variables. An independent ground-truth speed measurement for each overtaking vehicle was not available; therefore, direct external validation of the extracted speeds was not performed.

### 2.3. Variables of overtaking maneuver

**Fig 3:**
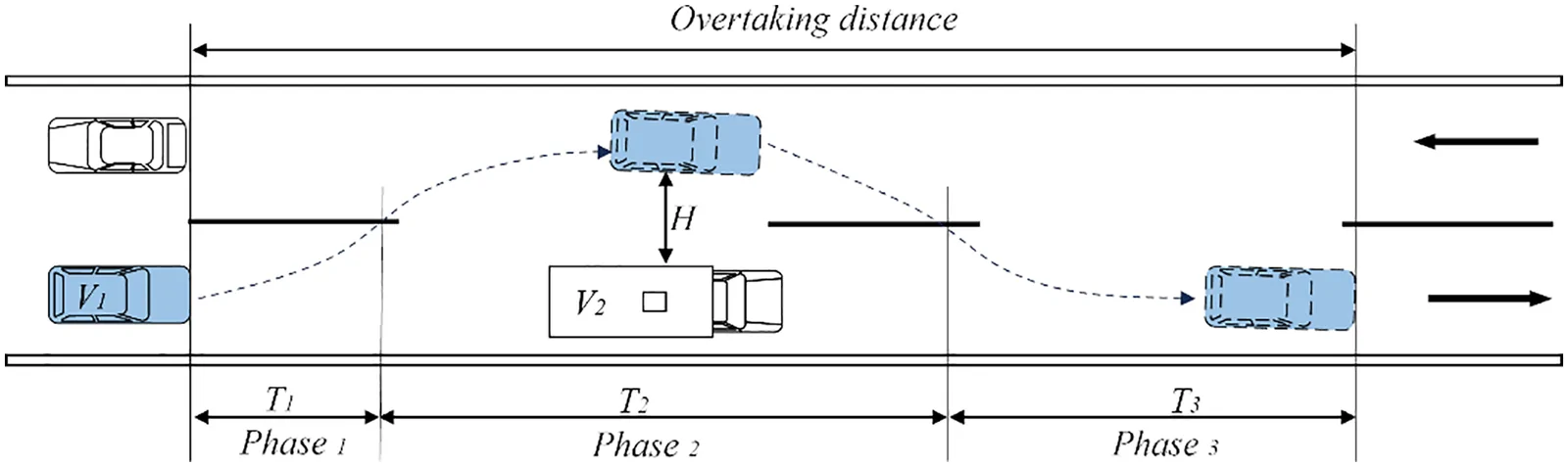
Phases of the overtaking maneuver.

As illustrated in Fig 3 and consistent with [20], an overtaking maneuver can be represented as three consecutive stages. Accordingly, the total overtaking duration equals the sum of the durations of these stages, i.e., ${\text{T}}_{\text{od}}{\text{=T}}_{\text{1}}{\text{+T}}_{\text{2}}{\text{+T}}_{\text{3}}$, where T_1_ represents the duration of the overtaking vehicle crossing the centerline (phase 1), T_2_ represents the duration of the overtaking vehicle occupying the opposite lane (phase 2), and T_3_ represents the duration of the overtaking vehicle crossing the centerline to return to the origin lane (phase 3).

Referring to studies on overtaking duration [4,17,20] and incorporating mixed traffic characteristics of two-lane highway. The candidate covariates for explaining overtaking duration are provided in Table 1.

**Table 1:** Variables of overtaking maneuvers.

Variable	Description	Units
Tod	Overtaking duration	Seconds
T1	Overtaking phase 1	Seconds
T2	Overtaking phase 2	Seconds
T3	Overtaking phase 3	Seconds
OD	Overtaking distance	Meters
ID	The initial longitudinal distance between the overtaking and the overtaken vehicles	Meters
FD	The final longitudinal distance between the overtaking and the overtaken vehicles	Meters
v1	The initial speed of the overtaking vehicle	Kilometer per hour
v2	The initial speed of the overtaken vehicle	Kilometer per hour
dv	dv = v1 − v2, initial speed difference between the overtaking and the overtaken vehicles	Kilometer per hour
H	The lateral distance	Meters
L	Length of the overtaken vehicle	Meters
Oncoming traffic	Having the oncoming traffic (0 represents without oncoming traffic and 1 represents with oncoming traffic)	
Overtaken vehicles	The type of overtaken vehicles (0 represents passenger cars(PC), 1 represents heavy vehicles (HV), 2 represents motorcycles (MC))	
Overtaking vehicles	The type of Overtaking vehicles (0 represents PC, 1 represents HV, 2 represents MC)	

## 3. Methodology

### 3.1. Survival analysis

Survival analysis provides a time-to-event modeling framework for data in which the response is the time elapsed until a target event occurs [21]. In this study, the framework is used to model overtaking duration as a time-to-completion outcome rather than as a failure process. The event of interest is defined as the completion of an overtaking maneuver, and the objective is to quantify how covariates are associated with this duration.

Let *T* denote the overtaking duration as a nonnegative random variable, and let f(t) denotes the probability density function of *T*. The distribution function of *T* can be expressed as:


$$\begin{array}{c} F\left(t\right)=P(T\leq t)=\int_{\text{0}}^{t}f\left(t\right)\text{d}t \end{array}$$ (1)

The survival function S(t) denotes the probability that the overtaking maneuver is not completed yet at time *t*, and is given by


$$\begin{array}{c} S\left(t\right)=P(T\geq t)=1-F\left(t\right)=\int_{t}^{\infty }f\left(t\right)dt \end{array}$$ (2)

To characterize the instantaneous completion tendency at time *t*, the hazard rate h(t) is defined by


$$\begin{array}{c} h\left(t\right)=\underset{\Delta t\to 0}{lim}\frac{P(t\leq T<t+\Delta t|T\geq t)}{\Delta t} \end{array}$$ (3)

A key feature of time-to-event data is censoring. In this study, censoring occurred when an overtaking maneuver was not completely observed within the UAV video window; in such cases, the exact completion time was unknown. These incomplete observations were treated as right-censored data. This formulation allows both complete and incomplete overtaking observations to be incorporated into the duration model.

### 3.2. Classical survival analysis model

Parametric survival models are commonly used to quantify the effects of explanatory variables on time-to-event outcomes. In this study, the Log-logistic accelerated failure time (AFT) model was selected as the parametric benchmark model. Unlike the proportional hazards (PH) model, which describes covariate effects on the hazard function, the AFT model directly relates covariates to survival time and is therefore suitable for interpreting how explanatory variables accelerate or decelerate the completion of an overtaking maneuver. The Log-logistic specification was adopted because previous overtaking-duration studies reported that it provided a suitable fit for overtaking-stage duration and mixed-traffic overtaking duration [17,20].

In the Log-logistic model, S(t) is represented using the covariates:


$$\begin{array}{c} S\left(t\right)=\frac{\text{1}}{1+exp(-\beta X)t^{\frac{\text{1}}{\gamma -1}}} \end{array}$$ (4)

and the hazard function h(t) is expressed as:


$$\begin{array}{c} h\left(t\right)=\frac{exp(-\beta X{)}^{\frac{\text{1}}{\gamma }}•t^{\frac{\text{1}}{\gamma -1}}}{\gamma (1+exp(-\beta X)t^{\frac{\text{1}}{\gamma }})} \end{array}$$ (5)

where *β* denotes the direction and degree of influence of the variable, X represents a vector of explanatory variables, and γ represents the shape parameter. In the AFT framework, the exponentiated coefficient exp(*β*) can be interpreted as an acceleration factor. A value of exp(*β*) greater than 1 indicates that an increase in the corresponding covariate is associated with a longer expected overtaking duration, whereas a value less than 1 indicates a shorter expected overtaking duration. The percentage change in expected overtaking duration associated with a one-unit increase in a covariate can be expressed as 100 × [exp(*β*) − 1] %.

### 3.3. The proposed survival machine learning analytical framework

**Fig 4:**
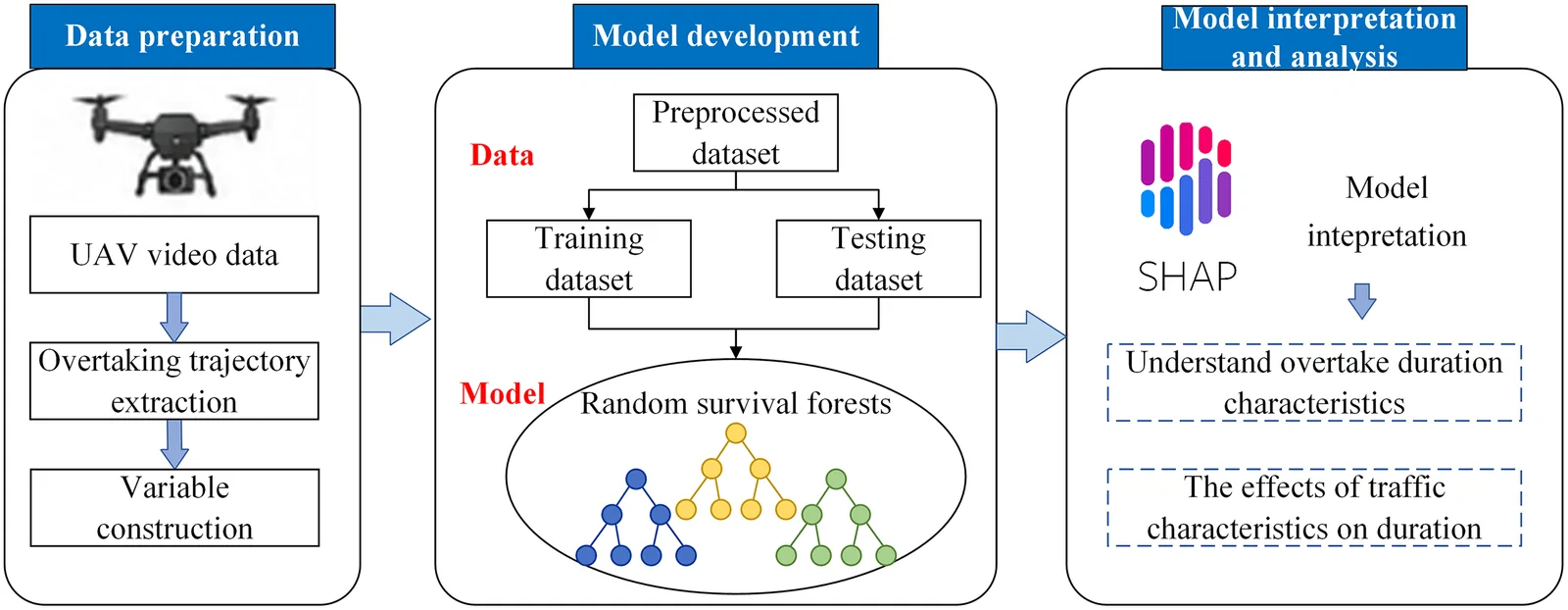
The proposed analytical framework.

A survival machine learning analytical framework was developed for overtaking duration analysis. As illustrated in Fig 4, the workflow includes data preparation, model development, and results interpretation. After dataset preprocessing, the overtaking duration model was built using RSF. Model performance was evaluated using the concordance index (C-index), and SHAP analysis was used to interpret how traffic and maneuver-related variables contributed to the RSF risk score.

#### 3.3.1. Random survival forest.

Random survival forest (RSF) was used as a non-parametric survival model because it can handle right-censored duration data and capture nonlinear effects and interactions without requiring a pre-specified survival distribution. As a well-established ensemble method for right-censored survival data, RSF is suitable for exploratory analysis with a limited sample size. These properties make it appropriate for modeling overtaking duration, where the effects of traffic and maneuver-related variables may be nonlinear.

Ishwaran et al. extended random forest (RF) to survival analysis and proposed the RSF method [22]. Similar to RF, RSF introduces the randomness in two main ways. First, samples are drawn from the original dataset with replacement to grow survival trees. Second, at each node, candidate split variables are selected from a randomly chosen subset of explanatory variables. Each survival tree estimates a cumulative hazard function (CHF), and the ensemble CHF is obtained by averaging the CHF estimates over all trees. The main steps for constructing the RSF model are as follows:

1. Draw bootstrap samples from the original data.
2. Build a survival tree for each bootstrap sample using a survival splitting rule.
3. Estimate a CHF for each tree.
4. Average the tree-specific cumulative hazard function to obtain the ensemble CHF.

#### 3.3.2. Interpretable machine learning framework.

To improve the interpretability of the RSF model, the SHAP method was used to quantify the contribution of explanatory variables to the RSF model output. SHAP is a widely used explainable machine-learning method based on Shapley values from cooperative game theory [23]. It measures the contribution of each feature by comparing the model prediction with and without that feature across different feature subsets. In this study, SHAP values were interpreted with respect to the RSF risk score derived from the ensemble cumulative hazard function. A positive SHAP value indicates an increased predicted tendency for earlier completion of an overtaking maneuver, whereas a negative SHAP value indicates a decreased predicted completion tendency.

Assume that the model input contains *n* features. For feature *i*, *S* denotes a subset of features that does not include feature *i*. SHAP calculates the contribution of feature *i* by averaging its marginal contribution to the model output over all possible feature subsets. The Shapley value of feature *i* is computed as:


$$\begin{array}{c} \phi_{i}=\sum_{S\in N}\frac{\left|S\right|!(n-|S|-1)!}{n!}\left[v\left(S⋃\left\{i\right\})-v(S\right)\right] \end{array}$$ (6)

where $\phi_{i}$ is the Shapley value of feature *i*, v(S) is the model output using the feature subset *S*, and v(S⋃{i})−v(S) represents the marginal contribution of feature *i*. A larger absolute SHAP value indicates a greater contribution of the corresponding feature to the model prediction.

### 3.4. Evaluation metric

Model performance was evaluated using the concordance index (C-index), a discrimination metric commonly used in survival analysis with censored observations [24]. The C-index measures the extent to which the predicted ordering of overtaking completion times agrees with the observed ordering. For both the log-logistic AFT and RSF models, prediction scores were defined such that higher values indicate a greater tendency for an overtaking maneuver to be completed earlier.

For two observations α and β, a comparable pair is formed when observation βhas a shorter observed overtaking duration than observation α($T_{\beta }<T_{\alpha }$) and the duration of observation βis observed ($\delta_{\beta }=\text{1}$). The pair is considered concordant if the observation with the shorter duration also receives a higher predicted ranking score, that is, $r_{\beta }<r_{\alpha }$. The C-index is calculated as:


$$\begin{array}{c} C=\frac{\sum_{\alpha ,\beta }{\text{1}}_{T_{\beta }<T_{\alpha }}\cdot {\text{1}}_{r_{\beta }<r_{\alpha }}\cdot \delta_{\beta }}{\sum_{\alpha ,\beta }{\text{1}}_{T_{\beta }<T_{\alpha }}\cdot \delta_{\beta }} \end{array}$$ (7)

where $T_{\alpha }$and $T_{\beta }$denote the observed overtaking durations of observations αand β, respectively;${\;r}_{\alpha }$ and $r_{\beta }$ are the corresponding predicted scores; 1(·) is the indicator function; and $\delta_{\beta }\;$ is the event indicator, with $\delta_{\beta }=\text{1}$ indicating a completed overtaking maneuver and $\delta_{\beta }=\text{0}$ indicating a right-censored observation.

The C-index takes values in [0.5, 1], where 0.5 indicates random ordering and 1.0 indicates perfect discrimination. Both the log-logistic AFT model and the RSF model were evaluated using the same metric; therefore, their comparison reflects differences in discriminative ability rather than direct prediction errors of overtaking duration.

## 4. Results and discussion

### 4.1. Descriptive analysis

The collected video dataset includes 1,361 vehicle trajectories. Trajectory points were extracted at an interval of 0.03 s, resulting in 544,400 trajectory observations. The traffic flow data showed an average daily traffic volume was 2984 vehicles/day. The mixed traffic flow has distinctive characteristics compared to homogeneous traffic flow. The observed traffic stream exhibited mixed-traffic characteristics, with passenger cars accounting for the largest proportion, followed by heavy vehicles and motorcycles.

**Fig 5:**
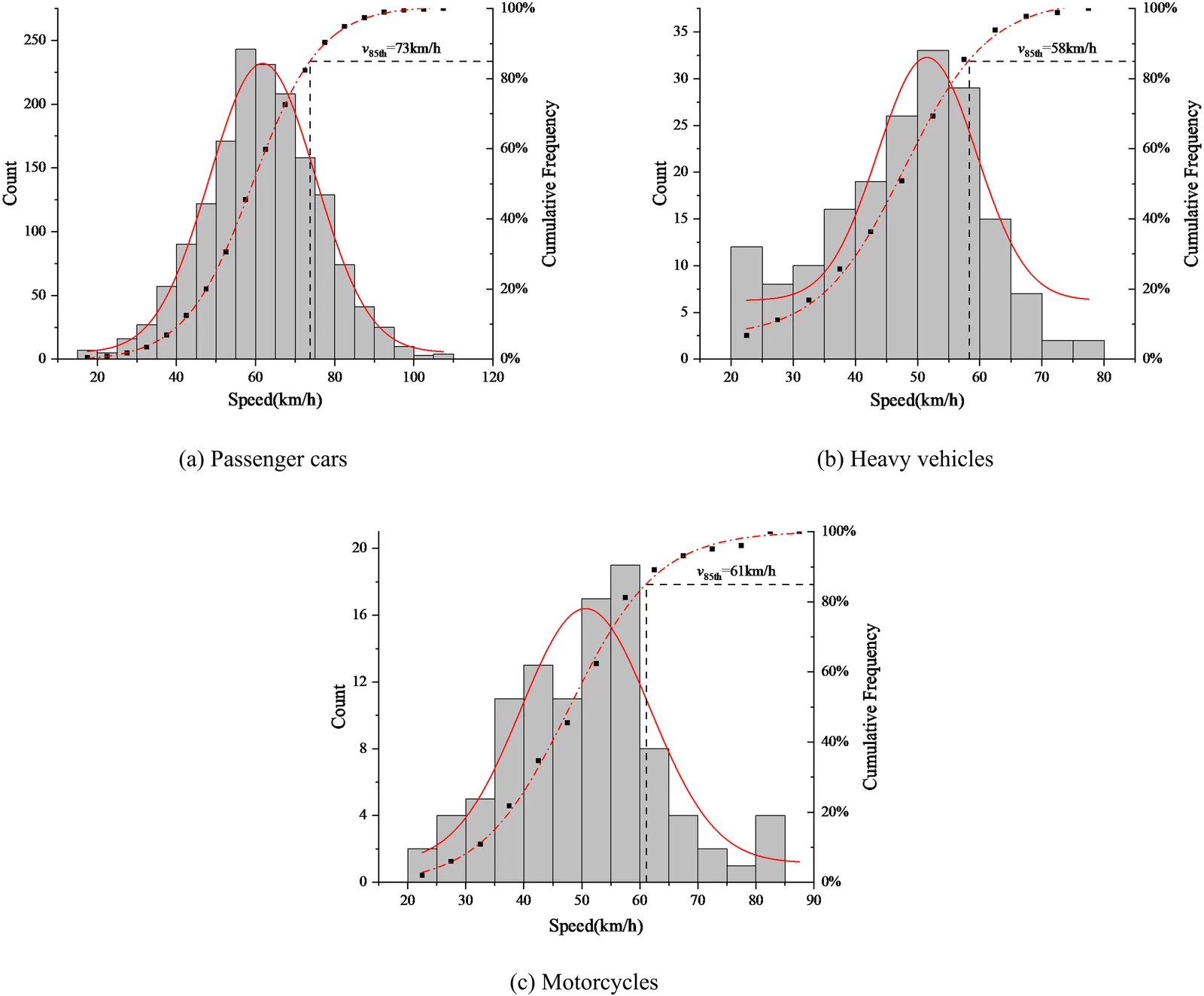
The speed distribution histogram and cumulative frequency curve.

Fig 5 shows the speed distributions and cumulative frequency curves of passenger cars, heavy vehicles, and motorcycles. The speed distribution of passenger cars was approximately normal, and the 85th percentile speed was 73 km/h, which exceeded the speed limit of 60 km/h. The speed distributions of heavy vehicles and motorcycles were more concentrated near the speed limit. These differences indicate speed heterogeneity among vehicle types, which may create overtaking opportunities on rural two-lane highways.

A total of 93 overtaking observations were included in the final survival-analysis dataset, including 73 complete overtaking maneuvers and 20 right-censored overtaking maneuvers that were not fully observed within the UAV video window.

Table 2 summarizes the vehicle-type composition in the traffic flow, among overtaken vehicles, and among overtaking vehicles. Heavy vehicles and passenger cars accounted for relatively high proportions of overtaken vehicles, whereas passenger cars were the dominant overtaking vehicles. No motorcycle was observed as an overtaking vehicle in the final dataset. This pattern suggests that motorcycles played a limited role as overtaking vehicles in the observed rural mountainous traffic environment, although this finding should be interpreted cautiously given the site-specific sample [20].

**Table 2:** Vehicle-type composition in traffic flow, overtaken vehicles, and overtaking vehicles.

Vehicles type	The percentage of vehicle types in the traffic flow	Vehicle type as the percentage of overtaken vehicles	Vehicle type as a percentage of overtaking vehicles
Motorcycles	11.1%	18.4%	0%
Passenger cars	58.3%	35.5%	84.2%
Heavy vehicles	30.6%	46.1%	15.8%

Table 3 shows the descriptive statistics of continuous overtaking maneuver variables. The phase durations (T1, T2, and T3), overtaking distance, and final longitudinal distance were calculated based on the 73 complete maneuvers, whereas variables available for both complete and right-censored observations were summarized using all 93 observations. The average overtaking duration and distance are 10.2 s and 201.2 m, respectively. Among the three phases, T2 had the longest mean duration (7.2 s), indicating that the passing phase accounted for the largest proportion of total overtaking duration. This result aligns closely with the 7.6 s reported in the field study by Llorca and García [25].

**Table 3:** Descriptive statistics of continuous overtaking maneuver variables.

Variable	Description	N	Mean	SD	Min	Max	85th	95th
Tod	Overtaking duration	73	10.2	2.4	5.0	15.6	12.5	13.6
T1	Overtaking phase 1	73	1.7	1.1	0.3	6.2	2.5	4.2
T2	Overtaking phase 2	73	7.2	2.2	3.4	13.5	9.4	10.6
T3	Overtaking phase 3	73	1.3	0.6	0.3	3.2	2.0	2.6
OD	Overtaking distance	73	201.2	51.1	76.4	308.4	252.0	273.4
FD	The final longitudinal distance between the overtaking and the overtaken vehicles	73	30.5	11.2	10.4	67.0	41.6	50.9
ID	The initial longitudinal distance between the overtaking and the overtaken vehicles	93	25.9	12.3	6.8	63.9	37.1	55.2
v1	The initial speed of the overtaking vehicle	93	68.2	12.1	40.4	97.3	81.3	87.7
v2	The initial speed of the overtaken vehicle	93	56.9	12.0	20.5	83.3	68.0	73.6
dv	The initial speed difference between the overtaking and the overtaken vehicles	93	11.3	11.7	−2.2	60.0	19.6	37.4
H	The lateral distance	93	3.8	0.4	2.6	4.6	4.3	4.5
L	Length of the overtaken vehicle	93	7	4.3	1.8	18.0	12.2	14.2

Table 4 reports the frequency distribution of categorical overtaking maneuver variables, including the presence of oncoming traffic, the type of overtaken vehicle, and the type of overtaking vehicle.

**Table 4:** Frequency distribution of categorical overtaking maneuver variables.

Variable	Category	Count	N	Percentage
Oncoming traffic	No	80	93	86.0%
	Yes	13	93	14.0%
Overtaken vehicles	PC	37	93	39.8%
	HV	41	93	44.1%
	MC	15	93	16.1%
Overtaking vehicles	PC	76	93	81.7%
	HV	17	93	18.3%
	MC	0	93	0.0%

**Fig 6:**
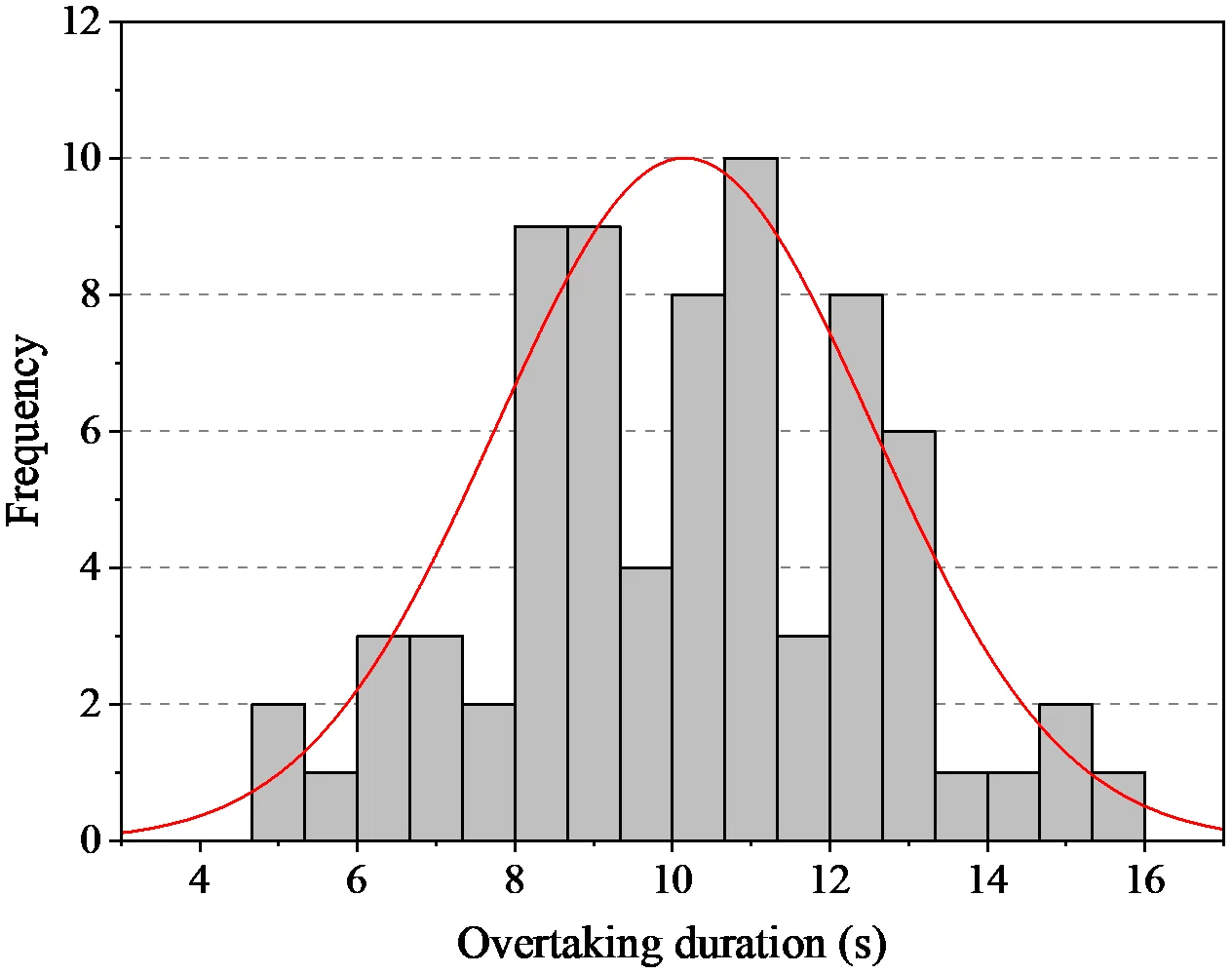
Distribution of overtaking duration.

Fig 6 depicts the distribution of overtaking duration. The mean overtaking duration and overtaking distance observed in this study were generally larger than those reported by Choudhari et al. under mixed-traffic conditions on two-lane highways in India [7], but were relatively close to the values reported by Llorca et al. for two-lane roads in Spain [25].

The mean initial longitudinal distance between the overtaking and overtaken vehicles was 25.9 m, which was larger than the average value of 13 m reported by Llorca et al. on two-lane rural highways in Spain [11]. The mean initial speeds of the overtaken and overtaking vehicles were 56.9 km/h and 68.2 km/h, respectively, indicating that overtaking vehicles often initiated maneuvers at speeds above the design speed of 60 km/h [25].

### 4.2. Model fit and performance comparison

The RSF model was developed using an 80/20 train–test split, with 80% of observations used for model fitting and hyperparameter tuning and the remaining 20% used for model evaluation. Four key hyperparameters were tuned: (a) the number of trees, (b) the maximum tree depth, (c) the minimum sample size to split an internal node, and (d) the minimum sample size in a terminal leaf.

Grid-search cross-validation was used to select the hyperparameter combination. During tuning, fivefold cross-validation was performed on the training set, and the mean C-index across validation folds was used as the selection criterion. The hyperparameter search space is shown in Table 5. Considering the limited sample size of the final survival-analysis dataset, the search space was designed to balance model flexibility and overfitting risk. The number of trees was varied from 50 to 300 to examine whether model performance stabilized as the ensemble size increased. The maximum depth and node-size parameters were tuned to control tree complexity and avoid overly deep trees.

**Table 5:** Hyperparameter search space for the RSF model.

Hyperparameters	Search space
Number of trees	50 to 300, step = 10
Maximum depth	2 to 30, step = 1
Minimum number of samples for splitting	2 to 20, step = 1
Minimum number of samples in a leaf	3 to 20, step = 1

Table 6 presents the top-ranked hyperparameter combinations obtained from grid-search cross-validation. The selected RSF model consisted of 150 survival trees, with a maximum depth of 8, a minimum of 7 samples required for node splitting, and a minimum of 10 samples in a terminal leaf. The model performance improved as the number of trees increased, but no substantial improvement was observed after approximately 150 trees. Therefore, the final configuration was selected by considering both predictive performance and model complexity.

**Table 6:** Best model configuration.

Rank	Number of trees	Max. depth	Min. no. of samples for splitting	Min. no. of samples in a leaf	Mean validation C-index
1	150	8	7	10	0.88
2	250	8	7	12	0.86
3	200	8	7	12	0.85
4	180	7	6	9	0.84
5	130	9	9	6	0.84

The log-logistic AFT model was fitted using the same survival-analysis dataset as a classical parametric benchmark. Model fitting was implemented in Python using the lifelines package, and parameters were estimated by maximum likelihood. Statistically significant variables were identified using backward stepwise regression with a significance threshold of p < 0.05 at each regression step. Model performance was evaluated using the C-index. After several trials, the optimal model was obtained as shown in Table 7.

**Table 7:** Estimates of the log-logistic AFT model.

Variable	β	SE	z	p-value	Exp(*β*)	95%CI	
						Lower	Upper
ID	0.007	0.002	3.314	<0.001	1.007	1.003	1.012
v1	−0.017	0.002	−7.714	<0.001	0.984	0.980	0.987
v2	0.013	0.002	5.402	<0.001	1.013	1.012	1.018
L	0.013	0.005	2.469	0.008	1.013	1.002	1.022
Oncoming traffic	−0.182	0.051	−3.297	<0.001	0.836	0.757	1.131
Overtaken vehicles	−0.049	0.027	−1.863	0.050	0.952	0.903	1.004
Overtaking vehicles	0.222	0.059	4.182	<0.001	1.227	1.124	1.356
γ	2.412						
Log-likelihood	82.73						
AIC	297.263						
C-index	0.768						

Note: ID, v1, v2, and L are continuous variables. Oncoming traffic, overtaken vehicles, and overtaking vehicles are categorical variables coded according to Table 1. γ is the shape parameter of the log-logistic AFT model.

The log-logistic AFT model and the RSF model were evaluated using the C-index. The C-index values were 0.768 for the log-logistic AFT model and 0.882 for the RSF model, indicating that the RSF model provided stronger discrimination of overtaking completion times in the present dataset. This improvement may be related to the ability of RSF to capture nonlinear associations and interaction effects among maneuver-related variables.

### 4.3. Model interpretation and feature analysis

#### 4.3.1. Effects of overtaking maneuver variables.

Previous studies have shown that maneuver-related factors are associated with overtaking duration [4,17]. However, the relative contributions of these factors may vary under rural mountainous traffic conditions and require further interpretation. In this study, SHAP analysis was used to interpret the trained RSF model and identify the main variables contributing to the model output. Because SHAP analysis explains the output of the trained RSF model, the direction of SHAP values should be interpreted with respect to the RSF risk score. A higher risk score indicates a greater tendency for earlier completion of an overtaking maneuver. Therefore, positive SHAP values indicate an increased predicted completion tendency, whereas negative SHAP values indicate a decreased predicted completion tendency.

**Fig 7:**
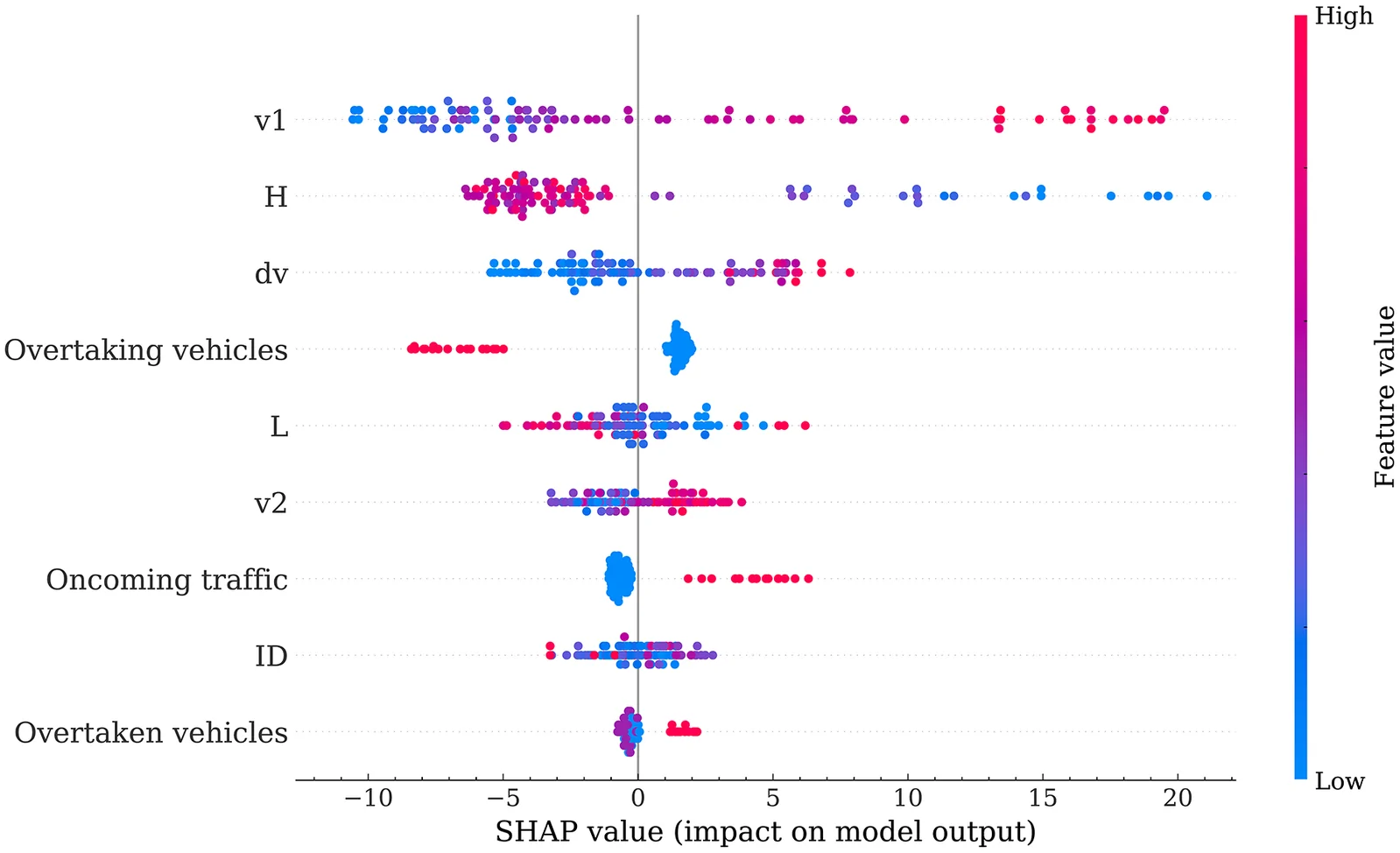
SHAP summary plot.

Fig 7 presents the SHAP summary plot of the RSF model. Each point represents one overtaking observation, and the color indicates the magnitude of the corresponding feature value. Features are ordered according to their mean absolute SHAP values, which represent their relative importance in the model. The main explanatory patterns identified from the SHAP results are discussed below.

The SHAP summary plot shows that the initial speed of the overtaking vehicle (v_1_) was the most influential variable in the RSF model. High v1 values were mainly associated with positive SHAP values, indicating an increased predicted completion tendency. This suggests that higher overtaking-vehicle speed is associated with earlier completion of the maneuver. In contrast, low V1 values were mainly associated with negative SHAP values, indicating a lower predicted completion tendency.

The initial speed difference (dv) showed a similar pattern. Larger speed differences generally increased the predicted completion tendency, whereas smaller speed differences were associated with a lower completion tendency. These results are consistent with the intuitive mechanism that higher overtaking speed and larger speed advantage can shorten the time required to complete an overtaking maneuver. However, a shorter overtaking duration should not be interpreted as universally safer, because high speeds may increase crash severity and reduce the time available for evasive actions [26,27].

The lateral distance (H) was also an important variable in the SHAP analysis. Larger H values were generally associated with lower predicted completion tendency, indicating that overtaking maneuvers with larger lateral separation tended to require longer completion time. This pattern may be related to differences in vehicle size, lane position, and lateral clearance during overtaking. Previous studies have also reported that larger lateral distance is associated with longer overtaking duration [4,28].

Vehicle-type variables also contributed to the RSF model output. Compared with PC, HV as the overtaking vehicle was associated with lower predicted completion tendency, indicating that overtaking maneuvers performed by HV tended to require longer completion time. This pattern is consistent with the operating characteristics of heavy vehicles, such as larger vehicle size and lower maneuverability. It also agrees with previous observations that overtaking maneuvers involving heavy vehicles require more careful timing and may be associated with higher operational risk [29]. The type of overtaken vehicle showed a smaller contribution than the type of overtaking vehicle, suggesting that overtaking duration was more strongly related to the characteristics of the overtaking vehicle in the present dataset.

The oncoming traffic variable showed a directional pattern in the SHAP summary plot. Observations with oncoming traffic were mostly associated with positive SHAP values, whereas observations without oncoming traffic were mainly distributed near or below zero. This indicates that the presence of oncoming traffic was associated with a higher predicted completion tendency in the RSF model. Compared with the variables discussed above, the contributions of L, ID, and overtaken vehicle type were smaller and more concentrated around zero, suggesting weaker overall effects on the RSF model output.

#### 4.3.2. SHAP dependence analysis.

**Fig 8:**
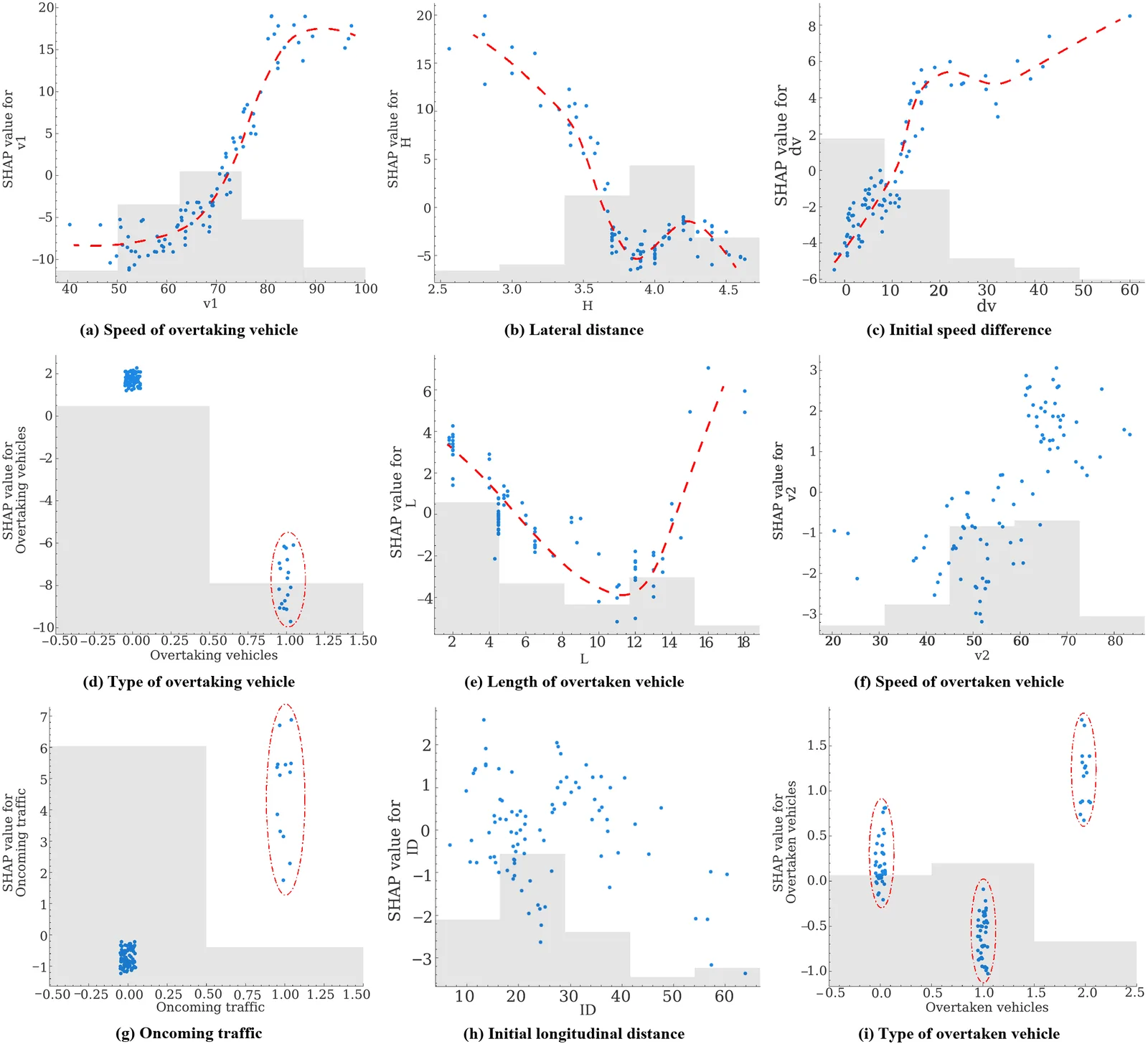
SHAP dependence plots of overtaking maneuver variables.

The SHAP dependence plots were used to further examine how individual variables contributed to the RSF model output (Fig 8). In each subplot, the x-axis represents the value of a given variable, and the y-axis represents the corresponding SHAP value for that variable. Positive SHAP values indicate an increased predicted completion tendency, whereas negative SHAP values indicate a decreased predicted completion tendency. The dashed horizontal line denotes a SHAP value of zero, the red dashed curve highlights the fitted trend, and the gray histogram shows the distribution of feature values.

For v1, the SHAP values were mostly negative below approximately 72 km/h and became positive after this threshold (Fig 8(a)). The fitted curve increased rapidly after v1 exceeded approximately 72 km/h and then gradually stabilized at higher speeds. This pattern suggests that a higher initial speed of the overtaking vehicle generally facilitates earlier maneuver completion, but the marginal contribution becomes less pronounced at very high speeds.

For lateral distance (H), the SHAP values were mostly positive when H was below approximately 3.6 m and decreased rapidly as H increased beyond this range (Fig 8(b)). Larger H values were mainly associated with negative SHAP values, indicating a lower predicted completion tendency. This pattern suggests that overtaking maneuvers with larger lateral separation tended to require longer completion time in the present dataset. This pattern may be related to differences in vehicle size and lateral clearance during overtaking [30].

For the initial speed difference (dv), the SHAP values increased rapidly as dv became larger (Fig 8(c)). When dv was small, most SHAP values were negative, indicating a lower predicted completion tendency. The fitted curve crossed zero at approximately 10 km/h and then remained at a relatively high level. This pattern suggests that speed advantage plays an important role in the completion of overtaking maneuvers.

The categorical variables showed category-specific patterns in the SHAP dependence plots. For overtaking vehicle type, HV observations were mainly associated with negative SHAP values (Fig 8(d)), indicating a lower predicted completion tendency than PC observations. This pattern is consistent with the operating characteristics of HV. In contrast, the type of overtaken vehicle showed a weaker contribution, with SHAP values concentrated closer to zero (Fig 8(i)). For oncoming traffic, observations with oncoming traffic were associated with positive SHAP values (Fig 8(g)), indicating a higher predicted completion tendency in the fitted RSF model.

The remaining variables showed weaker or less stable patterns. The length of the overtaken vehicle (L) showed a non-monotonic trend, with SHAP values decreasing at moderate L values and increasing again at larger values (Fig 8(e)). Because most observations were concentrated within a limited range of L, this trend mainly reflects the observed sample distribution. The effects of v2 and ID were less pronounced, with scattered SHAP values and no clear monotonic trend (Fig 8(f) and Fig 8(h)).

### 4.4. Discussion

The findings highlight the combined role of speed advantage and spatial interaction in overtaking behavior on rural mountainous two-lane highways. From a behavioral perspective, a larger speed advantage enables the overtaking vehicle to reach and pass the overtaken vehicle more quickly, thereby reducing the time spent in the opposing lane and shortening overtaking exposure. The threshold-like pattern observed for the initial speed difference (dv) further suggests that the effect of speed advantage is not constant across all operating conditions. Such nonlinear behavior may be difficult to fully capture using conventional parametric duration models with predefined functional forms, highlighting the value of flexible machine-learning-based survival approaches for overtaking-duration analysis.

The effects of lateral distance and vehicle type reflect the mixed-traffic characteristics of the study section. Spatial separation, vehicle size, and operating characteristics may influence the time required to complete an overtaking maneuver and the exposure to opposing traffic. In addition, the dominance of the passing phase (T2) indicates that vehicle interaction during this stage is central to overtaking exposure. Therefore, speed advantage, lateral clearance, and gap acceptance during the passing phase are particularly important for evaluating overtaking risk on rural two-lane highways.

The comparison with previous field studies further suggests that overtaking-duration characteristics are context dependent. The mean overtaking duration and distance observed in this study were generally larger than those reported by Choudhari et al. for mixed traffic on two-lane highways in India [7], but were relatively close to the values reported by Llorca and García for two-lane rural highways in Spain [25]. In addition, the larger initial and final longitudinal distances observed in the present study indicate that drivers on rural mountainous highways may initiate overtaking with a larger spacing margin and complete the maneuver with a longer return distance. These differences may be related to the mountainous road environment, mixed traffic composition, and regional driving behavior. Therefore, overtaking-duration models and safety countermeasures developed for non-mountainous or more homogeneous traffic conditions may not be directly transferable to rural mountainous two-lane highways.

The model comparison suggests that the RSF-SHAP framework provides useful complementary information to the log-logistic AFT model, particularly for identifying nonlinear and heterogeneous patterns. However, this comparison should be interpreted as dataset-specific predictive performance rather than evidence of the general superiority of RSF over classical survival models. The SHAP results should also be interpreted as model-based explanatory patterns rather than causal effects, especially because some variables, such as v1 and dv, are related by definition.

Several limitations should be acknowledged. The final survival-analysis dataset was relatively small and was collected from a single rural mountainous road section, which may limit generalizability. Although trajectory extraction was calibrated and visually checked, potential calibration and trajectory-extraction errors may propagate into the derived speed and distance variables. Roadway geometric characteristics and maneuver-level traffic-flow variables were not explicitly incorporated into the survival models. In addition, the analysis focused on total overtaking duration rather than modeling individual overtaking phases separately, and only a parametric AFT model and an RSF model were included in the primary comparison. Future studies using larger multi-site trajectory datasets could incorporate roadway geometry, short-interval traffic-flow indicators, phase-specific duration models, and additional semi-parametric survival models, such as the Cox proportional hazards model, to provide a broader benchmark for evaluating overtaking-duration modeling performance.

## 5. Conclusion

This study examined overtaking duration on rural mountainous two-lane highways using UAV trajectory data and an interpretable survival machine-learning framework. The observed average overtaking duration and overtaking distance were 10.2 s and 201.2 m, respectively. Among the three maneuver phases, the passing phase (T2) accounted for the largest proportion of total overtaking duration, indicating that this stage contributes most to overall overtaking exposure. Compared with the log-logistic AFT model, the RSF model showed better discrimination of overtaking completion times in the present dataset, suggesting that flexible survival machine-learning approaches can provide useful support for modeling overtaking-duration data.

The results indicate that overtaking duration is jointly influenced by speed advantage and spatial interaction between vehicles. The initial speed of the overtaking vehicle, speed difference, lateral distance, and overtaking vehicle type were identified as key factors, and nonlinear contribution patterns were observed for overtaking-vehicle speed and speed difference. These results highlight the importance of accounting for nonlinear and threshold effects when analyzing overtaking behavior.

This study also illustrates the value of survival-analysis frameworks for handling incomplete overtaking observations, which are common in naturalistic traffic data. The proposed framework provides a practical approach for analyzing censored overtaking-duration data and offers insights that can support overtaking safety assessment and traffic management on rural mountainous two-lane highways.
